# Involvement of Taiman in juvenile hormone signaling controlling sexual maturation in a male moth

**DOI:** 10.1016/j.cris.2026.100122

**Published:** 2026-01-16

**Authors:** Stéphane Debernard, Edmundo Gassias, Paleo Aguilar, Annick Maria, Annabelle Fuentes, Philippe Couzi, Françoise Bozzolan, Nicolas Durand, Evan Force

**Affiliations:** a Sorbonne Université, Université Paris-Est Créteil, INRAE, CNRS, IRD, Institute of Ecology and Environmental Sciences of Paris, 75005, Paris, France; b University of Madrid, Institute of Biology, Pozuelo de Alarcon, 28223, Madrid, Spain; c Sorbonne Université, Université Paris-Est Créteil, INRAE, CNRS, IRD, Institute of Ecology and Environmental Sciences of Paris, 78026, Versailles, France; d Université Paris-Saclay, UVSQ, INSERM, Infection et Inflammation, 78180, Montigny-Le-Bretonneux, France

**Keywords:** *Agrotis ipsilon*, Steroid receptor coactivator, Hormonal signaling, Sexual behavior, Accessory sex glands

## Abstract

In insects, juvenile hormone (JH) is essential for orchestrating reproductive events. For example, in the male moth *Agrotis ipsilon*, the behavioral response to female sex pheromone is linked to neuronal sensitivity in the primary olfactory centers (antennal lobes, ALs), and the maturation of accessory sex glands (ASGs) are known to be age- and JH-dependent. The molecular basis of this regulatory action of JH is not fully deciphered, and we show here that the heterodimerizing partner of Methoprene-tolerant called Taiman (Tai) is essential for the sexual maturation of male *A. ipsilon. Tai* expression in ALs and ASGs is elevated from the third day of adult life and is responsible for the acquisition of behavioral responsiveness to the sex pheromone and ASG maturation. *Tai*-deficient old males exhibited altered sexual behavior and delayed ASG maturation. Moreover, the expression levels of *Tai* and *Krüppel homolog 1* (*Kr-h1*), an early JH-induced transcription factor, were reduced in ALs and ASGs of JH-deprived and *Tai*-deficient old males, respectively. Exogenous JH injection into young males resulted in precocious sexual maturation and this JH induction was suppressed by *Tai* silencing. Our results demonstrate that Tai is an actor of the JH signaling pathway that operates in ALs and ASGs to promote pheromone information processing and consequently the display of sexual behavior in synchrony with ASG maturation, ultimately optimizing male reproductive success. Thus, this study provides additional insights into the molecular mechanisms underlying hormonal regulation of sexual maturation in insects.

## Highlights

• Tai mRNAs in ALs and ASGs are at higher levels in sexually mature old males than in sexually immature young males.
• Tai silencing in old males causes a decrease in sexual behavior.
• Tai-deficient older males exhibit delayed ASG development.
• JH deprivation by allatectomy results in a reduction of Tai expression in ALs and ASGs.
• Tai is a mediator of JH actions through a positive regulation of Kr-h1.

## Graphical abstract

**Figure:**
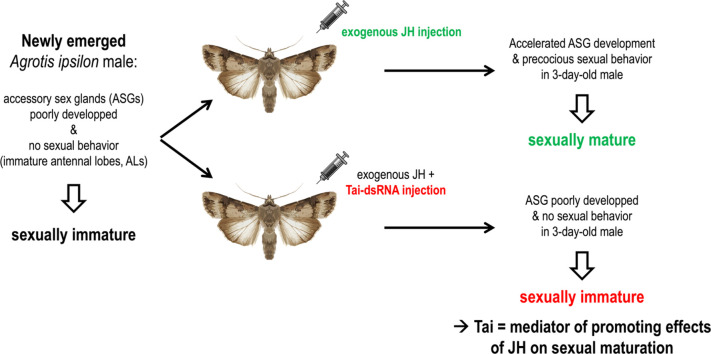

## 1 Introduction

Juvenile hormone (JH) is regarded as one of the most fascinating hormonal regulators in invertebrate endocrinology. In insects, it is produced by the *corpora allata* (CA), a pair of endocrine glands located close to the brain (Tobe and Stay, 1985). JH influences a wide spectrum of biological processes, including development, diapause, caste formation, migratory activity, lifespan, and reproduction (Flatt et al., 2005; Goodman and Cusson, 2012; Nijhout, 2021). During larval development, JH prevents the onset of metamorphosis by maintaining juvenile traits (Hiruma and Kaneko, 2013; Jindra et al., 2013), an effect achieved through its interplay with the ecdysteroid signaling cascade (Liu et al., 2018; Riddiford et al., 2003). In the adult stage, JH governs numerous reproductive functions and behaviors (Force and Debernard, 2025). For example, in females it supports previtellogenic growth in the yellow fever mosquito *Aedes aegypti* (Caroci et al., 2004), promotes vitellogenesis and oocyte maturation in the oriental fruit fly *Bactrocera dorsalis* (Yue et al., 2018), and contributes to reproductive regulation in the migratory locust *Locusta migratoria* (Wyatt and Davey, 1996). Moreover, JH modulates sex pheromone biosynthesis and female receptivity in the fruit fly *Drosophila melanogaster* (Bilen et al., 2013; Ringo et al., 1991). In males, JH has been shown to affect the physiology of accessory sex glands (ASGs) across different insect groups (Adiyodi, 1988; Duportets et al., 1998; Gassias et al., 2021; Gold and Davey, 1989; Kurogi et al., 2024; Parthasarathy et al., 2009; Ramesh et al., 2025; Wolfner, 1997). Administration of JH or its synthetic analogs has been reported to accelerate ASG maturation (Couche et al., 1985; Duportets et al., 1998; Force et al., 2024a; Parthasarathy et al., 2009; Regis et al., 1985). Additionally, in the noctuid moth *Agrotis ipsilon*, JH is essential for modulating central nervous processing of pheromonal cues that underlie male sexual behavior (Aguilar et al., 2023; Anton and Gadenne, 1999; Force et al., 2024a; Gadenne and Anton, 2000).

The intracellular receptor Methoprene-tolerant (Met) is a key mediator of multiple JH functions (Konopova and Jindra, 2007; Wilson and Fabian, 1986). Met is classified within the basic helix–loop–helix (bHLH)/Per-ARNT-Sim (PAS) family of transcription factors (Ashok et al., 1998). Its PAS domain, composed of two structurally related hydrophobic repeats, PAS-A and PAS-B (Kewley et al., 2004), serves as a crucial dimerization module. In vitro studies demonstrated that Met binds JH with relatively high affinity through a ligand-binding cavity formed within the PAS-B region (Charles et al., 2011; Miura et al., 2005). When JH is present, Met requires the partnership of another bHLH-PAS protein to assemble a heterodimeric complex that recognizes E-box motifs (JHRE) in the promoters of JH-responsive genes, thereby promoting their transcription (Li et al., 2014, 2011; Zhang et al., 2011). The partner of Met for the binding to JHRE is a coactivator related to vertebrate steroid receptor coactivators (SRC1/NCoA-1/p160), known in *D. melanogaster* as Taiman (Tai), in *A. aegypti* as Ftz-F1-interacting steroid receptor coactivator (FISC), and in other insects as SRC (Charles et al., 2011; Jindra et al., 2024, 2015; Li et al., 2014, 2011; Zhang et al., 2011). Moreover, *Krüppel homolog 1* (*Kr-h1*), a zinc finger transcription factor of the C_2_H_2_ type, has been identified as a direct early-response gene downstream of Met (Cui et al., 2014; Kayukawa et al., 2012). In L. *migratoria, Kr-h1* transcription depends specifically on the Met-Tai complex in response to JH. Knockdown of either *Met* or *Kr-h1* suppresses JH-driven *vitellogenin* (*Vg*) expression in the fat body, which in turn blocks oocyte maturation and ovarian growth (Song et al., 2014). Comparable RNAi-induced reproductive defects have been documented in *Met*- and *Tai*-depleted females of the bedbug *Cimex lectularius* (Gujar and Palli, 2016). Likewise, in the linden bug *Pyrrhocoris apterus*, disruption of *Met* or *Tai* halts ovarian development and significantly reduces *Vg* transcript levels in the fat body (Smykal et al., 2014). In *D. melanogaster*, Met has also been linked to male reproductive behavior by enhancing the responsiveness of olfactory receptor neurons to aphrodisiac pheromones (Lin et al., 2016). Collectively, these studies highlight Met as the primary regulator initiating JH signaling pathways that control insect sexual maturation.

Although the involvement of JH signaling pathway (comprising Met receptor and Kr-h1 transcriptional factor) in regulating reproductive system maturation and sexual behavior in insects is well-documented (Aguilar et al., 2023; Bebas et al., 2018; Gassias et al., 2021; Hartfelder, 2000; Holtof et al., 2021; Lin et al., 2016; Sláma, 2015; Wyatt and Davey, 1996), very little is known about the contribution of the co-receptor Taiman to this regulatory process in male insects. In fact, only one study by Holtof et al. (2021) has explored the involvement of Tai in the sexual maturation of adult male desert locusts, but it did not demonstrate its role in the maturation of the reproductive system or in the ontogenesis of sexual behaviors. Therefore, to further investigate this issue, we turned to the male moth *Agrotis ipsilon*, a model species for which we recently demonstrated that JH is involved in male reproductive development. Specifically, we showed that Met and Kr-h1 directly regulate sexual behavior in synchronism with growth and secretory activity of ASGs (Aguilar et al., 2023; Gassias et al., 2021). Upon adult emergence, male *A. ipsilon* are not yet sexually competent: their ASGs, a pair of elongated glands connected to the ejaculatory duct, remain undeveloped and contain little seminal protein (Duportets et al., 1998; Force et al., 2023a; Gassias et al., 2021). At this stage, males also fail to exhibit behavioral responses to female sex pheromones (Aguilar et al., 2023; Duportets et al., 1998; Force et al., 2024b; Gadenne et al., 1993). Under laboratory conditions, full sexual maturity is achieved roughly five days after emergence, at which point ASGs show increased seminal protein production (Duportets et al., 1998; Force et al., 2023a; Gassias et al., 2021). Mature males then respond to females by practicing oriented flights toward them, coinciding with the functional maturation of their antennal lobes (ALs), the primary olfactory centers (Aguilar et al., 2023; Duportets et al., 1998; Force et al., 2024b; Gadenne et al., 1993). Importantly, hemolymph JH titers are higher in older and sexually mature males than in newly emerged and immature ones, a difference linked in part to the age-dependent increase in JH synthesis by the CA (Duportets et al., 1998; Force et al., 2024a). In earlier work, we showed that silencing components of this signaling pathway, such as Met and Kr-h1, delays ASG maturation and suppresses behavioral response to the sex pheromone (Aguilar et al., 2023; Gassias et al., 2021). In light of our previous findings, particularly the direct involvement of Met in the sexual maturation of male *A. ipsilon*, and considering the well-established role of Tai as the partner of Met in the JH signaling pathway, we hypothesized that Tai would also be a crucial player directly responsible for male sexual maturation in insects by promoting *Kr-h1* expression in ALs and ASGs.

To delve the direct involvement of the JH co-receptor Taiman in the hormonal control of male sexual maturation in *A. ipsilon*, we successfully isolated a full-length cDNA fragment encoding a putative Tai protein and carried out a phylogenetic comparison of Tai across various insect orders. We next analyzed the tissue-specific distribution of *Tai* as well as its expression dynamics in ASGs and ALs throughout adult life. Furthermore, we assessed the consequences of RNAi-mediated silencing of *Tai* on the behavioral responses of 5-day-old males to female sex pheromones, and on the elongation and secretory activity of ASGs. To establish a link between Tai and JH action, we first tested how JH deprivation, induced by allatectomy or *Tai* knockdown, affected the expression of *Tai* or *Kr-h1* in ALs and ASGs of 5-day-old males. We then evaluated the effects of exogenous JH application, alone or in combination with Tai-dsRNA, on the initiation of sex pheromone-driven behavioral responses and ASG maturation in 3-day-old males. Altogether, our findings demonstrate that Tai participates in the JH signaling cascade regulating male sexual maturation by modulating ASG development and sex pheromone-guided orientation flight in the moth *A. ipsilon*.

## 2 Material and methods

### 2.1 Insects and tissue collection

Our study focused on newly emerged males of *Agrotis ipsilon* (Lepidoptera: Noctuidae). The insects were maintained under laboratory conditions at Sorbonne Université, France, and reared according to the procedure described by Force et al. (2023a). Each morning, freshly emerged males were transferred into transparent plastic containers (20 × 11.5 × 5 cm), with five individuals per box. The day of collection was considered the onset of adult life (day 1). Moths were supplied with a 12 % sucrose solution available ad libitum.

For the measurement of *Tai* and *Kr-h1* expressions, tissues were dissected during the middle of the dark phase (between 1:00 and 4:00 pm.), when the insects exhibit peak activity (Barrozo et al., 2010; Force et al., 2023b). Dissections were carried out under a Leica S9D stereomicroscope in Ringer’s solution (6.13 % glucose, 0.24 % HEPES, 0.11 % MgCl_2_, 0.07 % NaCl, 0.05 % KCl, 0.01 % CaCl_2_). Immediately following removal, the tissues were flash-frozen in Eppendorf (Montesson, France) tubes immersed in liquid nitrogen and stored at −80 °C until further use.

### 2.2 Allatectomy

Excision of the CA followed the procedure outlined by Gadenne et al. (1993). Males were subjected to allatectomy within three hours of adult emergence. Special attention was given to remove only the CA while leaving the *corpora cardiaca* intact. Control (sham) surgeries consisted of opening the head capsule and contacting the CA without excision.

### 2.3 Chemicals

At the onset of the scotophase, males were anesthetized with carbon dioxide, after which JH-II (15 μg dissolved in 2 μL of mineral oil) was administered into the lateral intersegmental membrane between the second and third abdominal segments using a 32-gauge Hamilton syringe (Reno, NV, USA). Control individuals received mineral oil injections only. The JH-II (78 % purity) was obtained from SciTech (Prague, Czech Republic).

In addition, a synthetic sex pheromone mixture was prepared at a concentration of 100 ng/µL. This blend consisted of three compounds previously identified from the pheromone glands of female *A. ipsilon* (Gemeno and Haynes, 1998; Picimbon et al., 1997): (Z)-7-dodecen-1-yl acetate (Z7–12:Ac), (Z)-9-tetradecen-1-yl acetate (Z9–14:Ac), and (Z)-11-hexadecen-1-yl acetate (Z11–16:Ac), combined at a 4:1:4 ratio. Field trials demonstrated that this composition was the most attractive to males (Causse et al., 1988), and wind tunnel assays confirmed that it triggers behavioral responses comparable to those elicited by natural gland extracts (Barrozo et al., 2010; Vitecek et al., 2013). The three pheromone components were purchased from Pherobank (Wijk bij Duurstede, The Netherlands) and diluted in hexane (> 98 % purity, CAS 110-54-3, Carlo-Erba, Val-de-Reuil, France). For wind tunnel behavioral assays, a 100 ng dose of the pheromone blend was applied, as this amount has previously been shown to be both electrophysiologically and behaviorally active (Deisig et al., 2014; Force et al., 2024b; Gadenne et al., 1993; Gassias et al., 2019).

### 2.4 Behavioral experiments to sex pheromones in wind tunnel

The study of behavioral response to female sex pheromones in male *A. ipsilon* was carried out using a Plexiglas® wind tunnel (VT Plastics, Genevilliers, France) measuring 188.5 cm (length) x 73 cm (width) x 72 cm (height). This device had 2 side hatches. Upstream of the tunnel was a filtration chamber consisting in a particle filter and an activated carbon filter. Downstream, the wind tunnel included an extraction hood, which generated an air stream in it by evacuating the air outside the building (0.3 m/s). In addition, at the downstream end of the device, a cage was positioned (28 cm high and at 36.5 cm width) to host the moth before the start of the experiment. At the other end, a glass vial containing 1 μL of odorant (deposited on a Wattman filter paper) was placed on a platform 26 cm above the tunnel floor and at 36.5 cm width; this vial was renewed every 4 moths (approximatively every 20 min) (for a diagram of the experimental setup, see Force et al. (2024b)). A Teflon tube linked to the vial was connected to a stimulation system composed of solenoid valves controlled by a ValveBank (ValveBankTM 4&8 II, AutoMate Scientific, Inc., Berkeley, USA). An electronic flow meter (Digital flow switch, PFM7 Series, SMC Corporation) set the flow rate of air entering the vial at 25 mL/min. The experiments were performed at a temperature and relative humidity respectively equal to 23 °C and 50–60 %. The room was into darkness and the wind tunnel was side-lit by an array of 11 infrared projectors (5 W, λ = 850 nm). The tunnel floor was covered with a black cloth.

Moths were transferred to the experimental room and left in darkness for at least two hours prior to testing to ensure proper acclimation. Behavioral trials took place between 13:30 and 16:00, corresponding to the scotophase when moth activity reaches its peak (Barrozo et al., 2010; Force et al., 2023b). For each trial, a single individual was introduced into the wind tunnel cage, where olfactory stimulation was immediately initiated and maintained for 3 min. Male moths were exposed to 100 ng of female sex pheromone. The sequence of tested individuals was randomized, and each insect was used only once. A 2-minute interval separated successive trials.

The male *A. ipsilon* behavioral response to the female pheromone is defined by an oriented flight, consisting of zigzagging movements upwind that extend beyond the midpoint of the tunnel. This behavior was recorded using 2 wide-field infrared-sensitive cameras (Basler Pilot Ace, piA640–210 m, equipped with Tamron 4–12F/1.2 lenses) positioned above the wind tunnel.

### 2.5 ASG length and protein content

The length of the dissected ASGs was determined visually with the aid of a microscope slide grid (1 mm × 1 mm) under a Leica S9D stereo microscope. Following this measurement, the ASGs were placed in 400 μL of 20 mM Tris buffer, pH 7.4 (Carl Roth, Karlsruhe, Germany), and homogenized for 20 s using a Polytron PT 10–35 GT homogenizer. The homogenates were subsequently sonicated and centrifuged at 10,000 rpm for 10 min at 4 °C in an Eppendorf Centrifuge 5804 R, after which the supernatant was collected. Protein concentration was assessed *via* the Bicinchoninic Acid (BCA) assay, employing a Bovine Serum Albumin standard curve ranging from 1 to 40 μg. For quantification, 10 μL of protein extract was combined with 10 μL of 20 mM Tris buffer (pH 7.4, Carl Roth, Karlsruhe, Germany) and 1 mL of working reagent, consisting of 20 μL of 4 % CuSO₄ and 0.98 mL of BCA solution. Samples were incubated at 37 °C for 30 min, and absorbance was measured at 562 nm using an Eppendorf BioPhotometer 6131 spectrophotometer. Each protein extraction data point was obtained from 3 pairs of accessory sex glands stored in 1.5 mL centrifuge tubes.

### 2.6 cDNA synthesis and cloning of *Tai*

Total RNA was isolated from pooled tissues of 5-day-old male specimens using TRIzol reagent (TRI Reagent®, Euromedex, Souffelweyersheim, France) following the manufacturer’s protocol, and RNA concentrations were determined with a UV/Visible spectrophotometer (NanoPhotometer® NP80, IMPLEN, Munich, Germany). Samples were treated with 2 U of TURBO™ DNase 1 (Ambion, Villebon-sur-Yvette, France) for 30 min at 37 °C, followed by enzyme inactivation at 75 °C for 10 min. Subsequently, single-stranded cDNA was generated from 1 μg of total RNA using SuperScript II Reverse Transcriptase (Invitrogen, Carlsbad, CA, USA) according to the manufacturer’s instructions. The reaction mixture included a dNTP mix, RNase OUT, Oligo(dT) primer, and nuclease-free water to reach a final volume of 20 μL. The mixture was initially heated to 65 °C for 5 min before adding the reverse transcriptase, and the reaction was then incubated at 42 °C for 1 h.

The amino acid sequence of *Helicoverpa armigera* Tai was used to query the *A. ipsilon* genome assembly in the NCBI database (accession GCA_028554685.1), and a primer pair was designed based on a predicted *AiTai* transcript. Polymerase chain reaction (PCR) was performed using 200 ng of cDNA with 2.5 U of High Fidelity DNA polymerase (Roche, Saint-Quentin Fallavier, France), 0.4 μmol/L of the primers (Taifor and Tairev), and 0.25 mmol/L of each dNTP. After an initial denaturation step at 94 °C for 5 min, amplification proceeded through 30 cycles consisting of 94 °C for 30 s, annealing at 55 °C or 57 °C for 30 s, and elongation at 72 °C for 2 min, followed by a final extension at 72 °C for 10 min. To capture the full-length *AiTai* cDNA, 5′- and 3′-rapid amplification of cDNA ends (RACE) were conducted using the SMART RACE cDNA Amplification Kit (Clontech, Mountain View, CA, USA) according to the manufacturer’s protocol. For 5′-RACE, specific reverse primers (Tai5’-RACE1 and Tai5’-RACE2) were used with the Universal Primer Mix (UPM, Clontech) as the forward primer, whereas for 3′-RACE, UPM served as the reverse primer with specific forward primers (Tai3’-RACE1 and Tai3’-RACE2). Touchdown PCR with hot-start was performed as follows: 1 min at 94 °C, 5 cycles of 30 s at 94 °C, 30 s at 67 °C, 1 min at 72 °C; followed by 5 cycles of 30 s at 94 °C, 30 s at 64 °C, 1 min at 72 °C; then 25 cycles of 30 s at 94 °C, 30 s at 62 °C, 1 min at 72 °C; and a final elongation for 15 min at 72 °C. All primers used for RT-PCR and RACE are provided in **Supplementary Table S1**.

RT-PCR and RACE products were first purified using agarose gel electrophoresis (NucleoSpin® Extract II, Macherey-Nagel GmbH & Co. KG, Düren, Germany) and subsequently cloned into the PCRII-Topo vector (Invitrogen). Following colony selection, plasmid DNA was extracted using a miniprep kit (NucleoSpin® Plasmid DNA Purification, Macherey-Nagel GmbH & Co. KG), and the clone carrying the correct insert was sequenced (GATC Biotech SARL, Marseille, France).

### 2.7 Expression quantification of *Tai* and Kr-h1

Total RNA was extracted from pooled tissues of multiple males (minimum of 15 individuals) and used for reverse transcription-quantitative polymerase chain reaction (RT-qPCR) with the LightCycler 480 Real-Time PCR Detection System (Roche Diagnostics GmbH, Mannheim, Germany), following the manufacturer’s protocol. Each 10 μL PCR reaction contained 5 μL of ABsolute Blue SYBR Green (Thermo Scientific, Waltham, MA, USA), 4 μL of cDNA (12.5 ng/μL), and 1 μL of 10 μmol/L gene-specific primers for either *Tai* (qTaifor and qTairev) or Kr-h1 (qKr-h1for and qKr-h1rev) (**Supplementary Table S1**). Amplification was performed over 50 cycles with the following conditions: 95 °C for 10 s, 65 °C for 15 s, and 72 °C for 15 s. The expected product sizes were 329 bp for *Tai* and 190 bp for *Kr-h1*.

Fluorescence analysis of melting curves within the 55–95 °C range revealed a single specific peak and confirmed the absence of primer-dimer artifacts for the primer sets employed. Each experimental run included a negative control (water) as well as a serial dilution of pooled cDNA (representing all conditions) prepared in five-fold steps. The dilution series (undiluted, 1/5, 1/25, 1/125, and 1/625) served to generate a relative standard curve, which was subsequently used to evaluate PCR efficiency and enable quantification. Across all assays, primer amplification efficiencies ranged between 95 % and 100 %. Reactions were performed in triplicate at the technical level and involved 6 independent biological replicates, using the facilities of the Institute of Ecology and Environmental Sciences of Paris (Sorbonne Université, France). Gene expression data were processed with the LightCycler 480 software (Roche Diagnostics GmbH) together with the GENORM Visual Basic tool for Microsoft Excel (Issy-les-Moulineaux, France), following the method described by Vandesompele et al. (2002). Threshold cycle (Ct) values were calculated for both the target genes and the reference gene encoding *ribosomal protein L8* (*RpL8*) of *Agrotis ipsilon* (accession number JX975720.1). *RpL8* displayed the most stable expression compared to other tested reference genes (*ribosomal protein L13, ribosomal protein S3, glyceraldehyde-3-phosphate dehydrogenase, α-tubulin*, and *β-actin*). The mean Ct of each triplicate was then applied to normalize candidate gene expression relative to *RpL8* using Q-GENE software (Simon, 2003) according to the following formula:

NE = (E RpL8)^Ct RpL8^ / (E candidate gene)^Ct candidate gene^

with NE meaning normalized expression, E meaning PCR efficiency, and Ct corresponding to the crossover point values. Subsequently, the mean and standard error were determined from the NE values obtained across the biological replicates. The sequences of the specific primers used for *RpL8* (qRpL8for and qRpL8rev) are listed in **Supplementary Table S1**, and the size of the amplified fragments was 200 bp.

### 2.8 RNA interference experiments

Conditions for the production and injection of double-stranded RNA (dsRNA) were adapted from protocols previously applied in *A. ipsilon* for *Tai* silencing (Aguilar et al., 2023; Force et al., 2024a). The dsRNAs targeting *Tai* (Tai-dsRNA, 614 bp) and the *bacterial β-galactosidase* gene (LacZ-dsRNA, 372 bp; used as control) were selected using the E-RNAi online platform (http://e-rnai.dkfz.de/) (Arziman et al., 2005) and synthesized with the MEGAscript® T7 High Yield Transcription Kit (Invitrogen), following the manufacturer’s protocol. PCR amplifications were carried out on 1 μL of plasmid DNA (50 ng/μL) using gene-specific primers (Tai-T7for/Tai-T7rev and LacZ-T7for/LacZ-T7rev; **Supplementary Table S1**). The cycling profile consisted of 35 repetitions of 95 °C for 30 s, 60 °C for 30 s, and 72 °C for 1 min. Amplified products were purified using the Nucleospin® Extract II Kit (Macherey-Nagel GmbH & Co. KG) and quantified on a UV/Visible NanoPhotometer® NP80 (IMPLEN). In vitro transcription was then performed overnight at 37 °C with 1 μg of purified PCR product in a 20 μL reaction mixture containing 2 μL of each nucleotide, 2 μL of T7 RNA polymerase, and 2 μL of 10X T7 buffer. After quantification, the mixture was treated for 15 min at 37 °C with 2 U of Turbo DNase (Invitrogen). dsRNA was precipitated by adding 30 μL of DEPC-treated water and 20 μL LiCl (Sigma Aldrich, Saint-Quentin Fallavier, France), followed by storage at −20 °C for 2 h and centrifugation at 16,000 g for 30 min. The resulting pellet was washed with 1 mL of 75 % ethanol, centrifuged, air-dried, and resuspended in 11 μL of DEPC-treated water. Denaturation at 95 °C for 5 min was followed by reannealing at room temperature for 1 h 30 min. The quality of dsRNA was verified by agarose gel electrophoresis, and prior to injection, samples were diluted to a final concentration of 0.5 μg/μL in saline solution.

Newly emerged males were anesthetized with CO₂ and injected with 1 μg of dsRNA into the lateral intersegmental membrane between abdominal segments two and three. Injections were performed at the onset of scotophase with a Hamilton syringe fitted with a 32-gauge needle. For 5-day-old males, ALs and ASGs were dissected for *Tai* expression analyses. Control treatments included uninjected males, saline-injected males, and males injected with bacterial LacZ-dsRNA.

### 2.9 Bioinformatics

The conserved domains (bHLH, PAS) of the *A. ipsilon* Tai (AiTai) protein were identified using the NCBI Conserved Domain Search tool (https://www.ncbi.nlm.nih.gov/Structure/cdd/wrpsb.cgi). Multiple sequence alignment for phylogenetic analysis was carried out with MAFFT version 7 (Katoh and Standley, 2013). A phylogenetic tree of insect Tai proteins was generated in MEGA 6.0 (Tamura et al., 2013) using the neighbor-joining approach with Poisson correction, applying default settings and 1000 bootstrap replicates. GenBank accession numbers for the Tai protein sequences included in this study are: lepidopterans *H. armigera* Tai (HaTai) ID: XP_049708301.2 and *S. exigua* Tai (SeTai) ID: CAH0700610.1; dipteran *D. melanogaster* Tai (DmTai) ID: AAG16637.1; coleopteran *T. castaneum* Tai (TcTai) ID: XP_008193622.1; orthopteran L. *migratoria* Tai (LmTai) ID: ANG56297.1; hymenopteran *A. mellifera* Tai (AmTai) ID: XP_006563176.2; crustacean *D. pulex* Tai (DpTai) ID: BAM83854.1; and human *H. sapiens* Tai (HsSRC1) ID: NP_003734. Pairwise sequence identity between AiTai and other insect Tai proteins was determined with the EMBOSS Needle web server (https://www.ebi.ac.uk/jdispatcher/psa/emboss_needle) (Madeira et al., 2024).

### 2.10 Statistical analysis

Data analysis was carried out with R version 4.1.2 (R Core Team, 2021) in RStudio 2021.09.2. Sample sizes are specified in the legends of the corresponding figures. The significance threshold was set at α = 0.05.

For behavioral assays, differences between groups of pre-exposed males were assessed using an *R* × *C* independence test, implemented through a G-test (a Chi-square test suitable for small sample sizes), with Williams’ correction for continuity applied (Sokal and Rohlf, 1995).

For datasets that did not meet normality assumptions according to the Shapiro–Wilk test, we used the Kruskal–Wallis test. When significant, post hoc pairwise comparisons were conducted using Dunn’s test.

### 2.11 Animal ethics

French animal welfare regulations are derived from European Union legislation (Directive 2010/63/EU), which grants ethical protection only to cephalopods among invertebrates. Nonetheless, all procedures were conducted with particular attention to minimizing stress and ensuring the animals’ well-being as far as possible

## 3 Results

### 3.1 Comparison of amino acid sequences and phylogenetic analysis of *Tai*

**Fig. 1:**
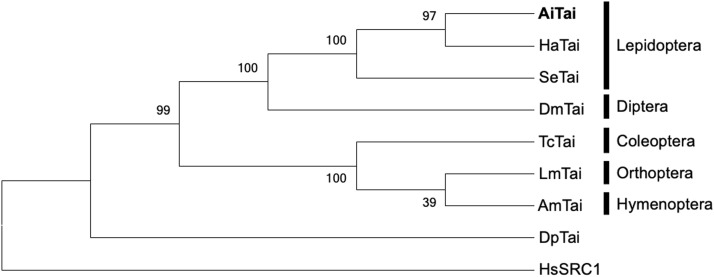
Neighbor-joining phylogenetic tree of Taiman (Tai) from *Agrotis ipsilon* and various insect orders. The number at each branch point represents the bootstrap probability. *Daphnia pulex* Tai (DpTai) and *Homo sapiens* Tai (HsSRC1) were used as outgroups to root the tree. The abbreviations and accession numbers of Tai sequences are given in the Material and methods section.

By searching the nucleotide sequence of *Helicoverpa armigera Tai* (phylogenetically closest species) against the genome database of *A. ipsilon*, we designed a pair of specific primers based on the sequence of a putative *Tai* transcript and succeeded in amplifying a partial fragment of 1513 bp *via* RT-PCR on brain total RNA extracted from 5-day-old males. The remaining 5′ and 3′ ends of this fragment were then obtained by RACE-PCR using gene-specific primers. The nucleic acid sequences for the 5′-RACE and 3′-RACE reaction products were assembled with the original fragment to generate a full-length cDNA named *AiTai*, which was deposited in the GenBank database under the accession number PV467640. The *AiTai* cDNA extends over 4908 bp and contains a 5′-untranslated region (5′-UTR) of 337 bp, an open reading frame (ORF) of 3782 bp, and a 3′-UTR of 789 bp with a polyadenylation signal upstream of the poly(A) tail. As expected, the analysis and comparison of the amino acid sequence of AiTai with insect Tai orthologs revealed the presence of the typical functional regions of the bHLH-PAS protein family (bHLH region was located at the N terminus and was followed by the PAS region) with a high percentage of identity, especially with the lepidopterans *H. armigera* (HaTai; 81.7 %) and *Spodoptera exigua* (SeTai; 75.4 %), followed by the coleopteran *Tribolium castaneum* (TcTai; 24.0 %), the hymenopteran *Apis mellifera* (AmTai, 20.6 %), the orthopteran *Locusta migratoria* (LmTai; 20.0 %), and the dipteran *D. melanogaster* (DmTai, 17.4 %) (**Supplementary Data S1**). A neighbor-joining phylogenetic tree of Tai was then created to examine the relationships of AiTai with Tai identified in other insect species (Fig. 1). The Tai tree was in concordance with the phylogeny of the represented insect orders, and as expected, its analysis showed that AiTai belongs to the cluster of lepidopteran Tai.

### 3.2 Tissue expression pattern of *Tai*

**Fig. 2:**
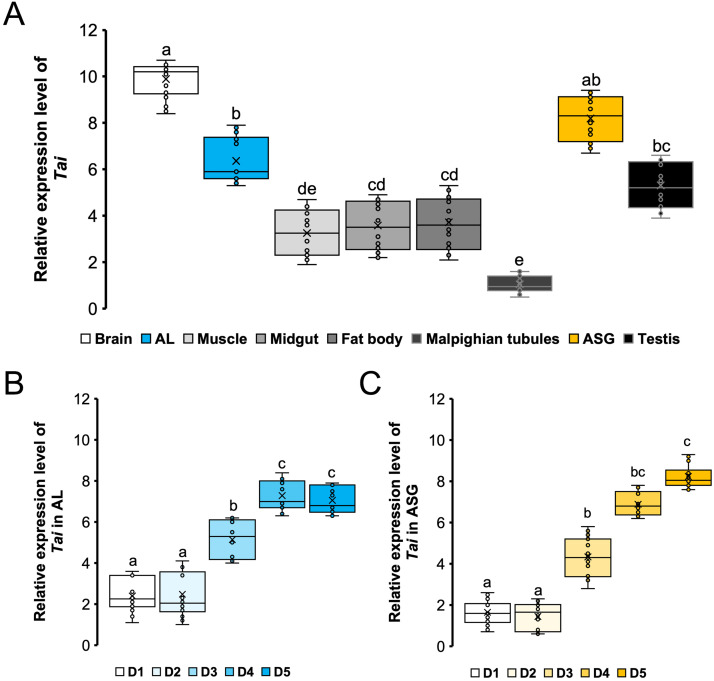
(**A**) Tissue expression pattern of *Tai* in 5-day-old *A. ipsilon* males. Total RNA from brain, antennal lobes (AL), thoracic muscles, midgut, fat body, Malpighian tubules, accessory sex glands (ASG), and testis were reverse transcribed to cDNAs for *Tai* expression analysis by qRT-PCR. The level of *Tai* transcripts in AL (**B**) as well as in ASG (**C**) were evaluated for 1- to 5-day old males. Each qRT-PCR was run in three technical replicates with six independent biological replicates. Crosses denote the mean, bars indicate the median, and boxes represent the 75 % quartiles; letters indicate statistically significant differences (Dunn's test, *p<0.05*).

To obtain preliminary information on Tai functions, the transcriptional activity of *Tai* was quantified by RT-qPCR in different tissues of 5-day-old males. As shown in Fig. 2**A**, compared to Malpighian tubules and muscles in which the level of *Tai* mRNA was low (Dunn’s test, *p* *=* *0.107*), *Tai* was highly expressed in the brain, ALs, and in ASGs (Dunn’s test, *p* *<* *0.001*), but also in the testes (Dunn’s test, *p* *<* *0.001*) and, to a lesser extent, in the midgut and fat body (Dunn’s test, *p* *≤* *0.026*).

### 3.3 Age-related changes in *Tai* expression within ALs and ASGs

To determine a possible role of Tai in sexual maturation, we quantified the amount of *Tai* transcripts in ALs (Fig. 2**B**) and ASGs (Fig. 2**C**) of males aged from 1 to 5 days after emergence. *Tai* mRNAs were present in ALs and ASGs from the first day of adult life and increased significantly from the day-3 (Dunn’s test, *p* *≤* *0.015*), then reached a maximum at day-5 (Dunn’s test, *p* *≤* *0.016*) (Fig. 2B & C).

### 3.4 Effect of *Tai* silencing on the behavioral response to female sex pheromone

**Fig. 3:**
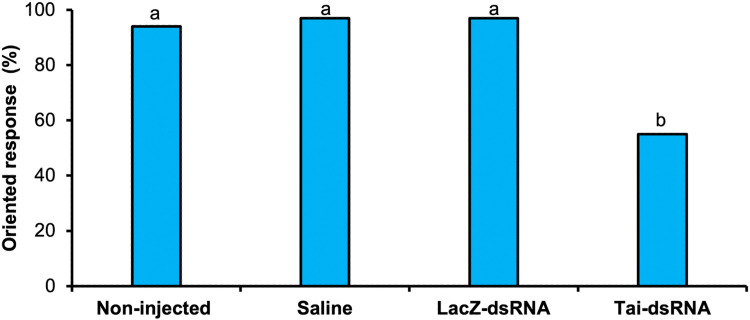
Effect of *Tai* silencing on the behavioral response of 5-day-old sexually mature males to sex pheromone. 1-day-old males received an injection of saline solution, bacterial β-galactosidase (LacZ)-dsRNA, Tai-dsRNA, or no injection (non-injected). For each treatment and four days after injection, the percentage of sex pheromone-triggered oriented flight was determined in the wind tunnel experiments (n_non-injected_ = 69, n_saline_ = 66, n_LacZ-dsRNA_ = 70, n_Tai-dsRNA_ = 65). Letters indicate statistically significant differences (G-test, *p<0.05*).

To test a potential involvement of Tai in the onset of sexual behavior, we analyzed the consequences of Tai-dsRNA injection in 1-day-old males on their behavioral response to female sex pheromone four days after treatment (Tai-dsRNA efficiency of 51 %, **Supplementary Fig. S1**). The 5-day-old males deficient for *Tai* showed a significant decrease in the display of sex pheromone-guided oriented flight (55 % for Tai-deficient males versus 95 % for control males) (G-test, *G* ≥ 10.33, *p* *<* *0.001*) whereas no significant difference was observed between the three control groups (G-test, *G* ≤ 0.05, *p* *≥* *0.828*) (Fig. 3).

### 3.5 Effect of *Tai* silencing on the growth in length and protein content of ASGs

**Fig. 4:**
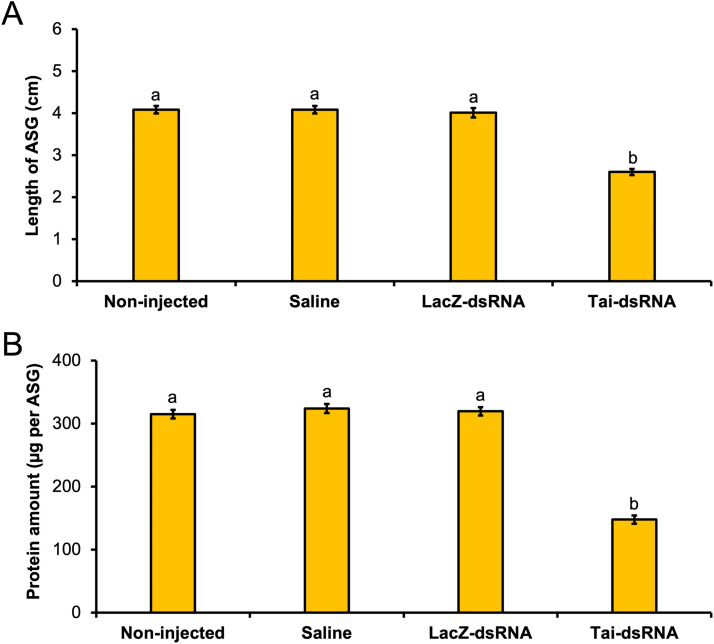
Effect of *Tai* silencing on the growth in length (**A**) and the protein content (**B**) of the ASGs. 1-day-old males received an injection of saline solution, bacterial β-galactosidase (LacZ)-dsRNA, Tai-dsRNA, or no injection (non-injected) and the length and protein amount of the ASG were determined four days after injection. For each experimental group, the ASG length was measured from 30 dissected glands and the ASG protein amount was quantified from three pairs of glands with eighteen biological replicates. The bars represent means ± sem; letters indicate statistically significant differences (Dunn's test, *p<0.05*).

In addition to its function in the onset of sexual behavior, we explored the putative action of Tai in the growth and synthetic activity of ASGs. To verify this, we examined the impacts of *Tai* silencing on the length and protein content of ASGs (Tai-dsRNA efficiency of 56 %, **Supplementary Fig. S1**). 1-day-old males injected with Tai-dsRNA showed a strong decrease in ASG length (2.6 cm for Tai-deficient males versus 4.0 cm for control males) (Dunn’s test, *p* *<* *0.001*) (Fig. 4**A**) and protein amount (148 μg per ASG for Tai-dsRNA injected males against 320 μg per ASG for control males) (Dunn’s test, *p* *<* *0.001*) (Fig. 4**B**) four days after treatment; whereas no significant difference was observed between the three control groups (Dunn’s test, *p* *=* *1.000*) (Fig. 4**A** & **B**).

### 3.6 Effect of JH deprivation on *Tai* expression and *Tai* silencing on Kr-h1 expression in ALs and ASGs of 5-day-old males

**Fig. 5:**
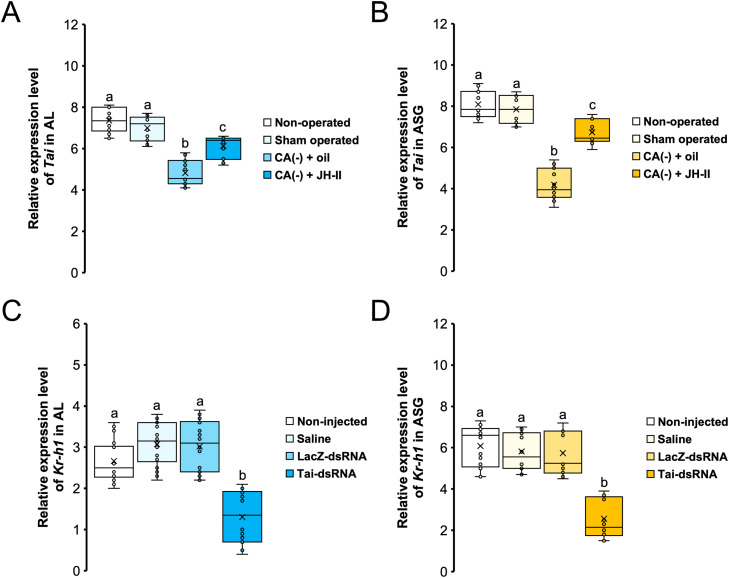
(**A-B**) Effect of allatectomy and JH-II injection on *Tai* expression in 5-day-old males. Allatectomy (CA(-)) was performed on day-1 and JH-II injection was carried out on the following day of allatectomy (day-2), and the relative expression of *Tai* in AL (**A**) as well as in ASG (**B**) were quantified at day-5. Controls groups were composed of non-injected and sham-operated. (**C-D**) Effect of *Tai* silencing on *Kr-h1* expression. 1-day-old males received an injection of saline solution, bacterial β-galactosidase (LacZ-dsRNA), Tai-dsRNA, or no injection (non-injected), and the expression level of *Kr-h1* were quantified in ALs **(C)** and ASGs **(D)** four days after injection. Each qPCR was run in three technical replicates and six independent biological replicates. Crosses denote the mean, bars indicate the median, and boxes represent the 75 % quartiles; letters indicate statistically significant differences (Dunn's test, *p<0.05*).

To reveal a potential relationship between Tai and JH action, we first manipulated circulating JH titer of 1-day-old males by the ablation of the *corpora allata* (CA(-)) and measured the transcriptional activity of *Tai* in ALs and ASGs four days after allatectomy. Allatectomized 5-day-old males exhibited a sharp decline in *Tai* mRNA levels in ALs and ASGs compared to control groups (non-operated and sham-operated) (Dunn’s test, *p* *<* *0.001*) (Fig. 5**A** & **B**). Then, we demonstrated that injection of JH-II, the following day of allatectomy, increased *Tai* mRNA expression in ALs and ASGs of 5-day-old allatectomized males (Dunn’s test, *p* *≤* *0.018*) at levels almost reaching those of non-operated and sham-operated males (Dunn’s test, *p* *≤* *0.042*) (Fig. 5**A** & **B**). Moreover, we showed that the injection of Tai-dsRNA into 1-day-old males resulted in an approximately 60 % reduction in *Kr-h1* mRNA expression in ALs and ASGs four days after administration (Dunn’s test, *p* *<* *0.001*) (Fig. 5**C** & **D**).

### 3.7 Effects of JH injection alone or combined with Tai-dsRNA on the sexual behavior and ASG maturation in 3-day-old males

**Fig. 6:**
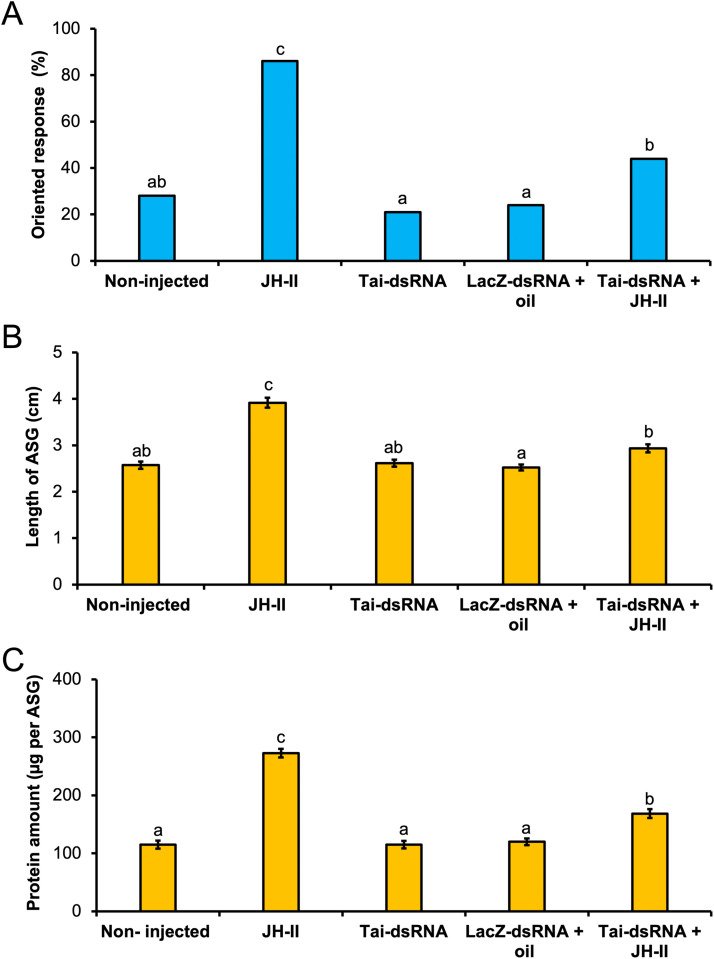
Effect of JH-II injection alone or combined with *Tai* silencing on the sexual behavior and the ASG maturation in 3-day-old males. JH-II and/or dsRNA injections were performed on day-1 and the behavioral response (**A**), as well as the length (**B**) and the protein content (**C**) of ASGs were analyzed at day-3. For (**A**), the percentage of sex pheromone-triggered oriented flight was determined in the wind tunnel experiments (n_non-injected_ = 68, n_JH-II_ = 65, n_Tai-dsRNA_ = 67, n_LacZ-dsRNA+oil_ = 70, n_Tai-dsRNA+JH-II_ = 71) and letters indicate statistically significant differences (G-test, *p<0.05*). For each experimental group in (**B**) & (**C**), the ASG length was measured from 30 dissected glands and the ASG protein amount was quantified from three pairs of glands with eighteen biological replicates; the bars represent means ± sem, and letters indicate statistically significant differences (Dunn's test, *p<0.05*).

To reinforce our previous results revealing the involvement of Tai in the JH signaling pathway controlling sexual maturation, we first injected exogenous JH-II in 1-day-old males and observed the effects on the behavioral response to sex pheromones as well as on the length and protein content of ASGs two days after treatment. Compared to untreated males, JH-II administration led to a precocious sexual maturation as early as the third day of adult life, as demonstrated by the increase in the sex pheromone-triggered oriented flight (G-test, *G* = 30.94, *p* *<* *0.001*) (Fig. 6**A**), as well as an increase in the length and protein content of ASGs (Dunn’s test, *p* *<* *0.001*) (Fig. 6**B** & **C**). Moreover, Tai-dsRNA administration coupled with JH-II injection in 1-day-old males suppressed the precocious induction of sexual maturation by JH-II at the third day of adult life. Indeed, in Tai-dsRNA and JH-II-treated males, behavioral responsiveness to the female sex pheromones (G-test, *G* = 3.56, *p* *=* *0.058*) (Fig. 6**A**), as well as the ASG length (Dunn’s test, *p* *≥* *0.093*) (Fig. 6**B**), were identical to untreated males; and the ASG protein content was inferior to JH-II injected males (Dunn’s test, *p* *=* *0.027*) (Fig. 6**C**).

## 4 Discussion

In this work, we tested the hypothesis that Tai directly contributes to the regulation of sexual maturation in male insects. The noctuid moth *A. ipsilon* was selected as a study model, since both the development of its sexual behavior and the maturation of ASGs are known to be influenced by age and JH (Aguilar et al., 2023; Gassias et al., 2021). As a first step, we successfully identified the nucleotide and amino acid sequences of Tai in *A. ipsilon* (**Supplementary Data S1**). These sequences display the conserved structural motifs typical of bHLH-PAS transcription factors, including the bHLH and PAS regions (Ashok et al., 1998; Charles et al., 2011; Kewley et al., 2004; Li et al., 2014, 2011; Miura et al., 2005; Zhang et al., 2011). Over the past decade, an explanatory framework has been proposed to describe the genomic mode of action of JH (Jindra et al., 2024, 2015; Tumova and Jindra, 2023). Within this framework, the bHLH domains of Met and Tai are considered key elements for dimer formation (Jindra et al., 2024, 2015). These domains are involved in folding and organizing α-helices, while the basic regions are thought to form the DNA-binding site required for the recognition of JH-specific response elements by the receptor complex (Jones, 2004; Li et al., 2014, 2011). In addition, the PAS domains of Met and Tai are presumed to participate in the dimerization process (Li et al., 2014, 2011). Consistent with these expectations, our phylogenetic analysis indicated that AiTai clusters within the lepidopteran Tai group, showing close relatedness to Tai from *H. armigera* and *S. exigua* (Fig. 1).

Moreover, although *Taiman* is a pleiotropic gene participating in multiple signaling pathways, including ecdysone, Hippo, and Hedgehog pathways, as well as potentially influencing circadian rhythms (Tumova et al., 2024; Zhang et al., 2015), we found that *Tai* exhibits differential expression across various tissues (Fig. 2**A**). Specifically, *Tai* shows high expression levels in the brain, ALs, and ASGs, whereas expression is lower in the testis, muscle, midgut, fat body, and Malpighian tubules. These observations suggest the presence of tissue-specific transcriptional regulation of the *Tai* gene. In addition, the tissue-specific expression pattern of *Tai* implies that JH primarily modulates functions within the nervous and reproductive systems, with Tai likely playing a key role in mediating these potential JH effects in adult male *A. ipsilon*.

Our findings revealed that *Tai* transcripts in ALs (Fig. 2**B**) and ASGs (Fig. 2**C**) are more abundant in sexually mature 5-day-old males compared with sexually immature 3-day-old males. This suggests an age-dependent increase in *Tai* expression in ALs and ASGs, coinciding with enhanced behavioral and neuronal responses to pheromone cues (Aguilar et al., 2023; Duportets et al., 1998; Force et al., 2024b; Gadenne et al., 1993), as well as with increased growth and biosynthetic activity of ASGs (Duportets et al., 1998; Force et al., 2023a; Gassias et al., 2021). These observations support the idea that Tai contributes to the development of sex pheromone-guided flight behavior and ASG maturation in *A. ipsilon* males. Consistent with this hypothesis, silencing *Tai* in older males disrupted oriented flight (Fig. 3) and delayed ASG growth (Fig. 4**A**), accompanied by reduced biosynthetic activity (Fig. 4**B**). Likewise, recent work demonstrated that RNA interference-mediated loss of *Tai* function impairs sexual maturation in *Schistocerca gregaria* males (Holtof et al., 2021).

**Fig. 7:**
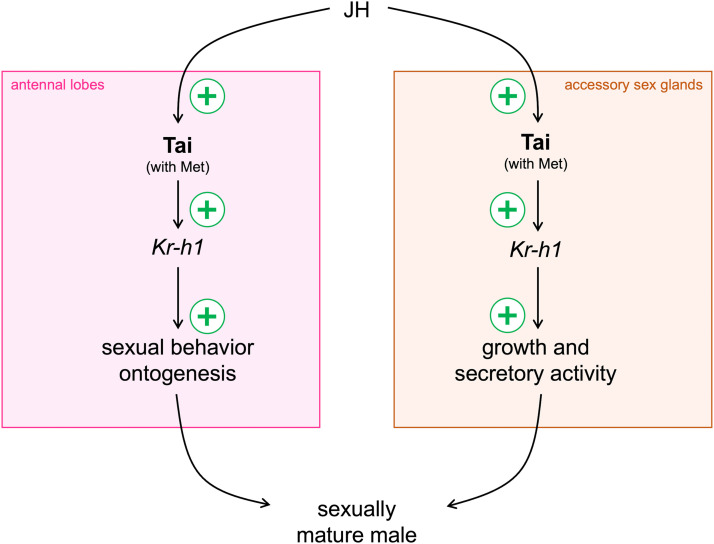
Diagram illustrating the juvenile hormone signaling pathway, involving the Taiman protein, that leads to sexual maturation in male moths of the species *Agrotis ipsilon*. JH: juvenile hormone; Tai: Taiman; Kr-h1: Krüppel homolog 1.

To establish a connection between Tai and JH activity, we observed that JH deprivation *via* allatectomy led to a marked decrease in *Tai* mRNA levels in the ALs (Fig. 5**A**) and ASGs of aged males (Fig. 5**B**). Similarly, we found that old males lacking *Tai* showed reduced *Kr-h1* mRNA expression in both ALs (Fig. 5**C**) and ASGs (Fig. 5**D**). Furthermore, elevating circulating JH-II levels in young males revealed that exogenous JH-II administration accelerated sexual maturation, as evidenced by an earlier onset of sex pheromone-directed behavior (Fig. 6**A**) and faster ASG development (Fig. 6**B** & **C**). Notably, these stimulatory effects of JH-II were blocked in *Tai*-deficient young males (Fig. 6). Collectively, these findings provide functional evidence that Tai directly mediates JH signaling in *A. ipsilon* males, promoting sexual behavior and ASG development through *Kr-h1* upregulation (Fig. 7). Considering our previous work demonstrating that the Met-Kr-h1-dependent JH pathway directly regulates male sexual maturation by delaying ASG development and diminishing sex pheromone responses (Aguilar et al., 2023; Gassias et al., 2021), Tai acts as a component of this JH signaling cascade probably through its dimerization with Met. Future studies could explore its interaction with Met isoforms (Met1 and Met2).

It would be valuable to demonstrate that Tai mediates the stimulatory effects of JH on the central processing of pheromone signals in the ALs, thereby facilitating the initiation of sex pheromone-induced oriented flight. To investigate this, we plan to assess how RNAi-mediated silencing of *Tai* influences the activity of pheromone-responsive neurons through intracellular recordings within the glomeruli of the macroglomerular complex in *A. ipsilon*, which are specialized for integrating pheromone information in the ALs (Deisig et al., 2014; Jarriault et al., 2010, 2009). Nevertheless, it remains possible that the proposed JH-Met-Tai-Kr-h1 signaling cascade operates in additional brain regions beyond the primary olfactory centers, particularly in second-order olfactory areas such as the lateral horn and mushroom bodies, which are known to exhibit substantial anatomical and functional plasticity in response to chemosensory experience and behavioral maturation (Menzel and Manz, 2005; Okada et al., 2007; Reinhard and Claudianos, 2012). Indeed, in situ hybridization studies in worker bee brains have shown that modifications in mushroom body synaptic architecture coincide with elevated *Kr-h1* expression during the transition to foraging behavior (Hewes, 2008; Shpigler et al., 2010). Furthermore, prior work has established that the antennal sensory system of *A. ipsilon* is fully operational in newly emerged males, with the electrophysiological responsiveness of pheromone-specific olfactory receptor neurons (Phe-ORNs) remaining stable regardless of age (Gadenne et al., 1993). This suggests that male sexual behavior in *A. ipsilon* is predominantly driven by central olfactory processing. However, studies in *D. melanogaster* and *A. ipsilon* indicate that JH can enhance sexual behaviors by increasing the sensitivity of antennal Phe-ORNs in sexually mature males, and that Met is critical for JH signaling (Force et al., 2025; Lin et al., 2016). Considering that Tai is the obligatory partner of Met for the binding to JHRE, it is plausible that Tai also contributes to the modulation of antennal pheromone detection by JH.

Studies in *Aedes aegypti* have provided in vitro evidence pointing to a potential interaction between the JH signaling pathway and JH-triggered phospholipase C-dependent inositol triphosphate signaling. This indirect crosstalk appears to promote Met phosphorylation through the activation of calcium/calmodulin-dependent protein kinase II and protein kinase C (PKC) (Liu et al., 2015; Ojani et al., 2016). It has been demonstrated that phosphorylation of Met enhances its association with Tai by disrupting Met-Met homodimerization and facilitating the binding of the JH-Met-Tai complex to the E-box within the JH response element (Jindra et al., 2024, 2015; Li et al., 2021). Consequently, this interaction modulates *Kr-h1* expression (Li et al., 2014, 2011; Zhang et al., 2011). Future investigations could explore whether a similar functional interplay between these JH-initiated signaling pathways occurs in the ALs and ASGs to regulate male sexual maturation in *A. ipsilon*. Supporting this notion, in vitro studies in *D. melanogaster* males revealed that PKC activation *via* phorbol ester treatment of ASGs led to enhanced protein synthesis (Yamamoto et al., 1988). Moreover, topical application of a JH analog was found to suppress ASG protein production in PKC-deficient mutants (Yamamoto et al., 1988). Taken together, these observations suggest that in *A. ipsilon* males, JH may stimulate ASG protein synthesis through a PKC-dependent pathway, potentially mediated by a membrane-bound JH receptor, which could act synergistically with the canonical JH-Met-Tai-Kr-h1 signaling cascade. To test this hypothesis, future experiments could examine the effects of calphostin C, a PKC inhibitor, on JH-induced *Met, Tai*, and *Kr-h1* expression, as well as on protein synthesis, in *A. ipsilon* ASGs in vitro.

JH is a key regulator of reproductive maturation and sexual behavior across numerous insect species (Bilen et al., 2013; Cusson et al., 1994; Holtof et al., 2021; Teal et al., 2000). In male *A. ipsilon*, JH promotes the development of the ASGs necessary for seminal protein production and modulates central processing of pheromonal cues underlying sexual behavior (Anton and Gadenne, 1999; Duportets et al., 1998; Force et al., 2024a; Gadenne et al., 1993). Our findings indicate that these effects of JH are mediated by the transcription factor Tai, likely through concurrent activation of the JH signaling pathway in both ASGs and ALs in response to rising JH titers. This interpretation is supported by age-dependent increases in CA biosynthetic activity (Duportets et al., 1998) and coordinated expression of *Met, Kr-h1* (Aguilar et al., 2023; Gassias et al., 2021), and *Tai* in ASGs and ALs during male sexual maturation. Elucidating the downstream target genes of Kr-h1 will be essential to fully understand this JH-driven reproductive coordination.

The fact that *Taiman*, a pleiotropic gene participating in multiple signaling pathways, including the ecdysone, Hippo, and Hedgehog pathways, and possibly influencing circadian rhythms (Tumova et al., 2024; Zhang et al., 2015), suggests a potential involvement in mechanisms operating in synchrony with the JH signaling pathway during sexual maturation in male insects. For instance, 20-hydroxyecdysone (20E) is known to promote the development of ALs, ultimately leading to the onset of sexual behavior in male *A. ipsilon* (Duportets et al., 2013). Since 20E binds to the ecdysone receptor (EcR), forms a heterodimer with its partner Ultraspiracle (Usp) (Riddiford et al., 2000), and, after replacing the corepressor SMRTER (Tsai et al., 1999) with the transcriptional coactivator Tai, activates the expression of target genes (Bai et al., 2000; Smykal et al., 2023), it is highly probable that Tai acts on male sexual maturation through the ecdysone signaling pathway in parallel with the JH signaling pathway.

## CRediT authorship contribution statement

**Stéphane Debernard:** Writing - review & editing, Validation, Supervision, Conceptualization. **Edmundo Gassias:** Validation, Investigation. **Paleo Aguilar:** Validation, Investigation. **Annick Maria:** Validation, Investigation. **Annabelle Fuentes:** Validation, Investigation. **Philippe Couzi:** Validation, Investigation. **Françoise Bozzolan:** Validation, Investigation. **Nicolas Durand:** Writing - review & editing, Formal analysis, Validation, Investigation. **Evan Force:** Writing - review & editing, Writing - original draft, Validation, Supervision, Formal analysis, Data curation, Conceptualization.

## Data availability statement

All data are contained within this article and in Supporting Information.

## Declaration of competing interest

The authors declare that the research was conducted in the absence of any commercial or financial relationships that could be construed as a potential conflict of interest.

## Appendix Supplementary materials

Image, application 1

Image, application 2

Image, application 3

Image, application 4
