# Supplementary figures and images for: Involvement of Taiman in juvenile hormone signaling controlling sexual maturation in a male moth
Source: Curr Res Insect Sci. 2026 Jan 16;9:100122. doi: 10.1016/j.cris.2026.100122 (PMC12859794; doi:10.1016/j.cris.2026.100122)

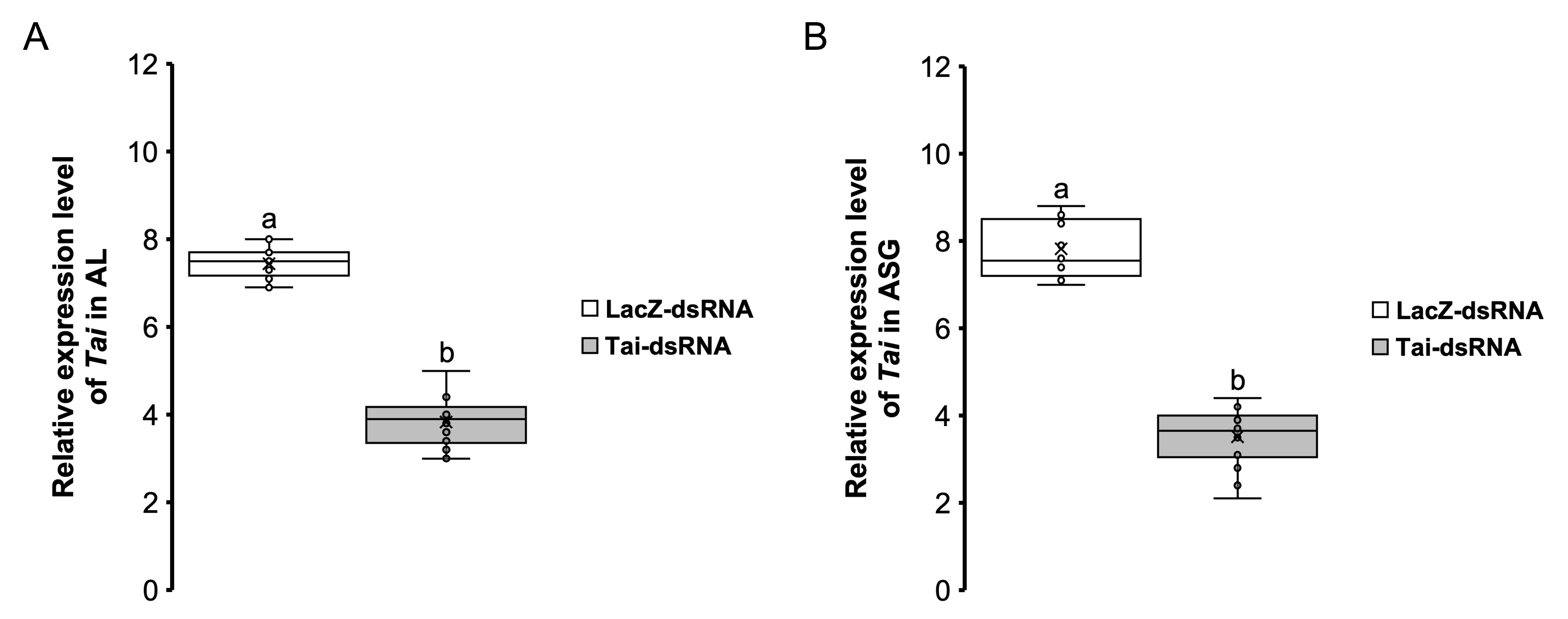

Supplement: Supplementary file 1 [file mmc1.zip › Supplementary_Figure_S1.jpg]
