# Supplementary material for: Involvement of Taiman in juvenile hormone signaling controlling sexual maturation in a male moth
Source: Curr Res Insect Sci. 2026 Jan 16;9:100122. doi: 10.1016/j.cris.2026.100122 (PMC12859794; doi:10.1016/j.cris.2026.100122)
Supplement: Supplementary file 2 [file mmc2.docx]

**Supplementary Table S1.** List of primers used in this study.

| **Name** | **Sequence** |
| --- | --- |
| Taifor  Tairev  Tai5’-RACE1  Tai5’-RACE2  Tai3’-RACE1  Tai3’-RACE2  qTaifor  qTairev  qKr-h1for  qKr-h1rev  qRpL8for  qRpL8rev  LacZ-T7for  LacZ-T7rev  Tai-T7for  Tai-T7rev | 5’-GAGCGAAACCGTCGTCTGGTGAGCC-3'  5’-GACGTCGGGCTGTTGCCGGTCTGCG-3'  5’-CCGAGGGTGCCGGTGTAATGCTTC-3'  5’-GGTCCGCCGACAGGAGGTCGAGGG-3'  5’-GCAGAGCGGCGGCCTGGCGGGCGG-3'  5’-CTCCACCCGCTGCAGCCGCCCGAC-3'  5’-GCGTGATCCACGCTGCGCCCGTGCG-3’  5’-CGAGTACCCACCAGGGGTATAGAAG-3’  5’-ACACACTGGTGAACGTCCGTAT-3’  5’-CGGTGTATCAGCGCCATGTTCTTT-3’  5’-CCAGTTTGTCTACTGCGGCAA-3’  5’-GCTTAACCCTAGTACGCTTGGCA-3’  5’-taatacgactcactatagggATGACCATGATTACGCCAAGC-3’  5’-taatacgactcactatagggCCATTCGCCATTCAGGCTGCG-3’  5’-taatacgactcactatagggCCCCGGCGACGGCGGCCCACCCGCC-3’  5’-taatacgactcactatagggGCTGCGACGGTCCCTCGACGGCGCTC-3’ |
