# Supplementary material for: Involvement of Taiman in juvenile hormone signaling controlling sexual maturation in a male moth
Source: Curr Res Insect Sci. 2026 Jan 16;9:100122. doi: 10.1016/j.cris.2026.100122 (PMC12859794; doi:10.1016/j.cris.2026.100122)
Supplement: Supplementary file 3 [file mmc3.docx]

AiTai ML---------PMVQTEPVLFNCGV--------------------------------------------------HSVNVAASPPATLPPSLTPESDV--

HaTai ML---------PMVQTEPVHFNCGV---------------------------------------------------SVNVAASPPATLPPSLTPDSDV--

SeTai ML---------PMVQTEPVLFNCGV--------------------------------------------------HSVNVAASPPATLPPSLTPDSEV--

DmTai M--------------SIAAAENAGLSPSDLP-------DHWASGTSNPSTSTSSSSSSSISASGSNNNKYNSGNISVCNLPRSSPAAATAAVTLSSAAGG

TcTai M------------------LEHSAMYLNVPPQY--------EDDTS------------------------------------------------------

LmTai M--------------STVIAEN------------------------------------------------------------------------------

AmTai MIAKRGKLDASARSEWRCVLEKIGK-------------ERWKNGRA---------------ARIGNEEKSGEEHWVRDNWVTAA----------------

AiTai ------------------------------DELVDIFFDVDVAVNTLPGIESCDLQ--------------------------------------------

HaTai ------------------------------DELVDIFFDVDVAVNTLPRIESCDLQ--------------------------------------------

SeTai ------------------------------DDLVDIFFDVDVAVNTVSRIESCDLQ--------------------------------------------

DmTai IGSNLLSGLHHGLAPGTHPLQRATAASGGGAGAGG--------VAGASTAAALTLQQHQHQQQQQQQQQQAQHQHQHQHQQQTQQQQLAYQHQQLQQQII

TcTai -----------------------------------FFPDF------YTRLGPCELQ--------------------------------------------

LmTai -----------------------------------------------AGLGPCDLQ--------------------------------------------

AmTai -------WIH--------------------DGGSD------------ARPSPRELQ--------------------------------------------

AiTai ----------------------------------LGDTWRGMQGKMSVTAPVKKIRKKSDNK---PQSQINKCHNEKKRRELENETINQLEELLGTCL-A

HaTai ----------------------------------LGDTWRGMQGKMSVTAPVKKIRKKSDNK---PQSQINKCHNEKKRRELENETINQLEELLGTCL-A

SeTai ----------------------------------LGDTWRGMQGKMSVPAPVKKIRKKSDNK---PQSQINKCHNEKKRRELENATINQLEELLSTCL-A

DmTai SHRSIQNKAAATAAAQTANLAAALQRSAAIMNAVASPISANSA---NSATSGRKIRRKTDSKVNLPQSQINKCNNEKRRREAENGYIEQLSEILTLNKRG

TcTai -----------------------------------DPAWAKMS---ALATGVNKKRKKSETK---PQAQINKCNNEKRRREQENIYIEELAELISANF-A

LmTai -----------------------------------DPLWVKMSA--AVTGGASKKRKKTDTK---SSAQVTKCLNEKRRRDQENTFYEELAELIAASF-A

AmTai -----------------------------------DPLWVKMS---AITGSISKKRKKSDAK---PQSQINKCLNEKRRRNQENLFIDELAELISA---T

bHLH

AiTai EVK----QPDKNGIVREATRQIQEVLRRRRE--------------------------------CPDECPLRASQC-------LSP-VQGGEVSSTQPSCA

HaTai EVK----QPDKNGIVREATRQIQEVLRRRRE--------------------------------CPEECPLRVAQC-------LSP-VQAGEVSSTQPSCA

SeTai EVK----QPDKNGIVSEATRQIEEVLRRRKE--------------------------------CPEECPLRAAQS-------RSP-VQAGEVSSTQPSCA

DmTai DMT--STKPDKAAILNQVVRTYREICDKGQNRDISST-STNNNNSTTTTNNNTNS-NNNNNTSKPQATSTRCSRCATD-NCSIHP-VQQGEVSSTEPPLP

TcTai DMSSLSVKPDKCAILQETVNQIRSIKQRESASQSSDP-------------------------------------------------VQQGEVSSSRPTIL

LmTai DMNSLSVKPDKCAILQETVTQIRRIKDQEGA--SSDA-------------------------------------------------IQQGEVSSSKPTIL

AmTai DMS--SGKTDKCQILQRTVDQIRHIREQEGS--NSHA-------------------------------------------------VQQGEVSSSNPNIL

AiTai -----GLHYSELASLIEALKHYTGTLGWVLLEINSEAKIECITENIKELTLQDRNELYDKSIFSLLHVKDHAKLRPLLRNIQ------------------

HaTai -----GLHYSELTSLIEALKHYTGTLGWVLLEINSKAEIECITENIKELTLQDRTELYKKSIFSLLHVKDHVKLRPLLRNIQ------------------

SeTai -----GLHYSETNSLIEALKHYTGTLGWVLLEINSKAEIVCITENIKELTLQDRTELYKKSIFSLLHSKDHAKLKPLLRSTQ------------------

DmTai EPSLLLGQVPEISAYFEALEHYISGVGWVLLQVNANGIIESCTQNIRDLIGYEKQELYHQPLYMYLYSGDHAKLEPIINTMYNNPNGGNSNSANNSGPGG

TcTai SNEVYG------PLLLEALEGF-------LFVVNAEGKVEHVTENVSNYIKFTRDEIFGNSIYNFIHLGDHARFTTSLMPM-------------------

LmTai ANEVLG------PLLLQALDGF-------LFIVNKDCHVEYVSENVEQFIKYTKEDILGKSIYNFMHVGDTTRFSSILLPIT------------------

AmTai SNDQVG------PILLEALDGF-------LFVVNTEGRVEYVTDNITQYINYTKDDVLGKDIYNIIHHGDHNTFMPSLLPM-------------------

AiTai ------TFNWGSGEI----------------------------DKFRAIQARLLVKNSNGTDSAG----------------FVECVIHAAPV--------

HaTai ------TFNWSSGEI----------------------------DKFQAIQARLLIKNSNGTDSAG----------------YAECVIHAAPV--------

SeTai ------NFNWGSSEI------------------------KTGQEKFQAIQARLLVKNSNGTDSTG----------------YVDCVIQAAAV--------

DmTai SSAGTSAGVWGDLEELNNGNASQGSNSSGAGGLGGAGGAAAGKKRSISTKVRMLVKDTRTATQTSSNCEEKPLRQSGHQDKYEEVVLIAAPV--------

TcTai -------IGWGSEST--------------------------TTTRSRSFSVRFLVKPPDDQDETV---EEKQQRVN----CYELMHISSTQL---RDQ--

LmTai ------STAWSGEH---------------------------QPHRNRTINCRLLIKPPDDADETM---EEKQQRIS----KYEYMQISSTLLPYPSDR--

AmTai ------QLGWTNEQ---------------------------QPQRNRTFNCRFLVKPPDDKEETM---EEKQQRVS----KYESMQICSALLPNNSDR--

AiTai -------------------------------------RGSSSEEAGSVMCVIRRCEDASAALLPGDGGPPAITAKH-SDHIVFRLDCNFIILFCDLSGVE

HaTai -------------------------------------RGSSSEEAGSVMCVIRRCEDASAALLPGDGGPPAITAKH-SDHIVFRLDCNFIILFCDLSGVE

SeTai -------------------------------------RSSSSEEAGAVMCVIRRCEDASAALLPGDGGPPASTAKP-SDHIVFRLDCNFIILLCDLRGVE

DmTai --------------------------------------KDDADASSSVLCLITRPEDESPLEI---NIQQHVQQQP-IEQMTFKLDIHGKILTLDPTALR

TcTai ----------------------VSVSD-----------DDGADSGPYLLCVASRISHRDKVI----SG---------IEQFTTKLDTSGKIIGVDASGVS

LmTai ----------------------SESGDL----------SESIDSSPCLMCVARRIPQNEKSL-----------TNP-VEQFSMKLDVHGKIVGVDTTNIS

AmTai ----------------------LESGDV---------SSESSDNGPCVMCVARRIPPNEKPI-----------GTP-IEQFTVKLDTTGKIIAVDVIWLS

PAS

AiTai NLINTSIP---LVGTRYLELVENS--DRIRVAAHLQEAVCLPAPPA-----------ISEPFRLRIAPDRPCFRVVARSRLFRAKP-SSGEPDFIMSTHT

HaTai NLINTSKP---LVGTRYLELVENC--DRIRVAAHLQEAVCLPAPPA-----------ISEPFRLRIAPDRPCFRVVARSRLFRAKP-SSGEPDFIMSTHT

SeTai NLINTAVP---LVGTRYLELVESS--DRIRVAAHLQEATCLPAPPA-----------ISEPFRIRIAPDRPCFRVIAQSRLFRAKP-SSGESDFIMSTHT

DmTai EPFKQHL-QT-WVGRLWQDLCHPH--DLSTLKSHLRDI--QDSASANSPGAGAGTSVVSRPFRLRLGAPDVYVHVKANSRLFLNQ--TPGEGDFIMSVQT

TcTai APYSQFINKE-LMDRALRDLVFQQ--DVHKLNTHLKET--IHTGQA-----------TSAVYRLQLGQ-DKYVQVQTKSKLFKTNPHNSNDIDFIMATHS

LmTai PSYSQFLNKD-LTGRVIQDLCHQQ--DLQKLTSHLREA--VQTGHN-----------TSKIYRLRVKTPDKYIQVQTKSRLFKN---PNDQEDFIMATHS

AmTai SPYSEYLSKE-LIGTAIKDLCHPH--DLNKLTAHLNDT--LQVGES-----------TSGVYRLRVSP-DKFLNIQTKSKLFKANVMNTLVTDFIMATNT

AiTai VLSDDDM-DLLENDS-----------------------------------------------------------------------PRPPVGGPLMTSV-

HaTai VLGDEDM-DLLETDG-----------------------------------------------------------------------PRPPVGGPLMTSV-

SeTai VLNDDDM-DLLESDG-----------------------------------------------------------------------PRPPVGGPLMTSV-

DmTai LLNSENDMNSSNTGAGSGGLGLGQLCAMAPSPSLASSLLSSLSMDGLHGGTGSGSSSSPAASGMLPTHLLGGLVGGGQQGGGGNSTQTTSVGGPLMSSAI

TcTai IVGEYDATGPSDPGG----------------------------------------------------------------------GGTSSVGGPLMTSV-

LmTai IIGDSEN-PLPECGH------------LSNSPR-------------------SATSAGQGNPSF----------------GTSPSDGNGSVGGPLMSSA-

AmTai IIGDNDL-TPIEGGQ------------LSNNKVC------------------SGHSSNRCANNS----------------NNNNGNNNNNVGGPLMSAVA

AiTai -------------ANGESSNCEPRYRSPISPSD-G-----------------------------------------------------------------

HaTai -------------ANGESSTCEPRYRSPISPND-GN----------------------------------------------------------------

SeTai -------------ANGESSSCEPRYQSSISPND-G-----------------------------------------------------------------

DmTai -------------ING--TGLQQQQQQQQRSGA-SSSA------------------------------------------SSSANALVNAFTASPAPAE-

TcTai -------------VNGTRNGTAPVSSGSDSSTA-------------------------------------------------SSNALMNAGNASFTPYI-

LmTai -------------VNGQVSAVGSVGGRTNSSGF-GSV---------------------------------------------TSDANSPAYHTYSTEIG-

AmTai -H-----------LNGQVSGMSSRGLAGTSSHA-GTAG------------------------------------------ATPSNSIVFSAAESCNPLPS

AiTai --------------PFLNDFDLEPWASSLLG--------------EMPSE--------------DTKERKESAVEG---PSQ---------PLTPRAPS-

HaTai --------------PFLSEFDLEPWASSLLG--------------EMPSE--------------DSKDRKESVPET---HSQ---------PLTPRAPP-

SeTai --------------TFLSEFDLEPWASSLL---------------DMPSE--------------ETKERKESV-EG---PSQ---------PPTPRAPS-

DmTai ---------HSFYGSDTFEFDIAAHSSSFEL---------------DPSG-GVGAW---------TDSRPNSRASVA-TPVS-----------TPRPPS-

TcTai --------------DNDFPFDIFP-TSTFEL---------------EPSG-----W---------TEARPDSRQSA--TPVS---------TPTPRPPS-

LmTai ----------------LNDFDFFP-SSTWDIVGAGETSPDGSSVNATQSG-----W----------ERRPDSRQSQSLTPVS---------TPNPRPPS-

AmTai LTTTSNPFNH-FSGNMDLEFELFP-SSTWDL--------------DSSSG-----W----------ADRPESRASG--PPNS-RPSS----QPAPTSPS-

AiTai TPGEGPPSN--------------------------------------------QP---------------------------------------------

HaTai TPGEGPPSN--------------------------------------------QP---------------------------------------------

SeTai TPGEGPPSN--------------------------------------------PP---------------------------------------------

DmTai GHGFSPAV-------C--ASP-ATPYQLSSHSAASLP----------------SP-QSNASAGGGNYGGFNFH----------------SFDSSDVKPE-

TcTai NPAYSPAT-----TVA--QSP-LAPFVTQP-----------------------SP--STPAAPNPYTSTFSFSPLSEQNYN-------------------

LmTai GAGYSPSG-----GVC--ASP-LNPYVHQT-----------------------SPAARVSTPAHPFANSFPFSPLQEQGPD-------GSFCSDG-----

AmTai PQGTFSNS-----AVAPHCSP-LRAF---------------------------SP--TSANAAHTFSNSFSLSPLQESTSSLTNSAASSSVSANGAAPGL

AiTai --------------------------------------------------------------------------------AEEPNRLRSLL---------

HaTai --------------------------------------------------------------------------------AEEPNRLRSLL---------

SeTai --------------------------------------------------------------------------------AEEPNRLRTLL---------

DmTai ------------KD---------VQQQQNQQQSNNNSSSS----NPLLGGGLPNGVGGMLLSQQQQQQQTPPQQQQQQQQQESSERLRHLL---------

TcTai ------------------------------------------------------------VEEPKDTKANVLDEASSM--MADSARLRNLL---------

LmTai -------------------------TEESKSVVQESANVN----GSSDSGSTP---------------------------GDSGRRLRNLLLT-------

AmTai TPKRQDEG----KSS-----AAGCPTTANQSVIEGNNGRT----NPASVAGTP------AVAQPPQTTATVVETQNSVV-STESGRLRNLL---------

LxxLL

AiTai -----------------------------SKKPMPGN-----------NDATVNSNNRILKDLLKQEDEDATGSETSAPHTPH------TPMTPHTPNAA

HaTai -----------------------------SKKPMPGN------------EGAVNSNNRILKDLLKQEDEEATGSETSAPHTPHTPHTPLTPHTPHTPSAA

SeTai -----------------------------SKKPMPAN------------DNNVNSNNRILKDLLKQEDEDATGSETSAPHTPHTPHTPHTPHTPHTPSAA

DmTai -----------------------------TKSQSMAGGLGG-----------LGDDEKYFKPEGSEEEKHASGGFKMG----------------GQPGGM

TcTai -----------------------------TKPPTVADS--------SANDADGRNKNRILKGLLNQQDEDDNRNDNRA-----------------SPRG-

LmTai -----------------------------KRQSTSADEVSAADAGGQGGDVDVRNRHRILKGLLNQDDDEESRGDE-------------------VPTGA

AmTai -----------------------------TKGGSASEE----TQDNTSNDTESQNKHRILKILLNQQDEDDFHPEHNNK-------------VRTSPS--

LxxLL

AiTai LSPLHT-QAHARPPP--HPPHAPLAQHAPHVSHASHVSHGSNTT----HTMQQAHHNNSDVLLRILNDKSDEDAEE-RRNSADGNRGVSQPSALLSQLLS

HaTai LSPLHS--AQPRPPP--HALHAPHQPHQPHPA-------------------PQHHHNNSDVLLRILNDKSDEDAEENRRNSGDGNRGVSQPSALLSQLLS

SeTai LSPLHTAAAHPRPQPHAHPAHAPHAAHPAHPPHPAHPAHSAHSAHPAPHSMQQPHHNNSDVLLRILNDKSDEDAEENRRNSADGNRGVSQPSALLSQLLS

DmTai -GMFGPMGSMGR-------------------------------GVGNSSMLHKAGNSQNPMLLKLLNEKSEDDDGN-GSGGGPGSMNNSRQSELMRQLKN

TcTai -GLISRGAVAG--PS----------------------------ELPKTS-----TAGGNNMLLQLLNERSDDDD------NLEARAGVRKKSELLHQLLK

LmTai -GAAGRGSVGGRLQT----------------------------DAPRMP---GHSSTGNDMLLKLLNEKSDDDD-------VEKRAGLKKQNELLQQLLK

AmTai -------------------------------------------NMPKPSMEHSKSSLGNNMLLQLLNEKNDDED-------EEARAGLKKRNELLQQLLK

LxxLL

AiTai SSNGPSGN-----------------------------GRQQES--------------------SENYLERIAGVKRKFEE--------------------

HaTai SSNGPSGN-----------------------------GRQQDN--------------------SDNYMERMAGVKRKFEE--------------------

SeTai SSNGPSGN-----------------------------GRQQES--------------------SDNYLERIAGVKRKFEE--------------------

DmTai PDGGSHGM-----------------------------HRNSASGNMS----------------TED---------------------------LKAML--

TcTai TESRENED-----------------------------EKKNDVQS------------------HDDSLLRSLGFTS-SPS--------------------

LmTai EQDEESKP-----------------------------QVGPDTGQH-------------R---DDDPLLESLGFRNPSPS--------------------

AmTai DQDDERKV-----------------------------QEQHCKPQ--------------R---EEDSLLRNLGFRNTTPS--------------------

AiTai --------GNKGVPT--------------------------------------NSKRATPEN-QQVTSSALPA---------------------------

HaTai --------TNKGAPS--------------------------------------NPKRATPEN-QQVTSSALPA---------------------------

SeTai --------A-KGVAN--------------------------------------NSKRATPEN-QQVTSSALPA---------------------------

DmTai --------KIQSDPS-------------------------------------LNRKRSLNEP-DDDPSAKR-----------------------------

TcTai --------PPSGEPG-------------------------------------RSRKRPSDDR-DENVSSKR-----------------------------

LmTai --------PPSGAGVGVD-----------------------VGGNLTVSSLIGGRKRASDEG-DESGVSSAKI-----------------RL--------

AmTai --------PSQS------------------------------GDNVGHSSSQVGQKRPGEDG-DLNIAAKRPM---------------------------

AiTai SSTASSTATSPATSSSP--------------------------------------------------------------GMSPLCQKNQILV--------

HaTai SSAASSTATSPATSSSP--------------------------------------------------------------GMSQLCQKNQILV--------

SeTai SSTTSSTATSPASSSSP--------------------------------------------------------------GMSSLCRKNQILV--------

DmTai --------------SED--------------------------------------------------------------KPSKLCTQNKMLA--------

TcTai -----TSDGSQVSSTGA------------G-------------------------------------------------TGSKLCEKNKMLA--------

LmTai SGPSGTPQGASRSGTAA--------------------------------------------------------------GTSKLWERNKMLA--------

AmTai ---DGSHQVSSSGTSTN--------------------------------------------------------------ATSKLWERNKMLA--------

Lx

AiTai ---SLLARQQ-----------TTPTTPLPL--------PNPNLRAYGPTARARPPPPPQPQMP----------PQRHLHSTLSNILTGANHRANSMNGGG

HaTai ---SLLARQQ-----------TTPTTPLPL--------PNPNLRAYGPTARPRPPPP--PQMP----------PQRHLHSTLSNILTGANHRPNNMNGGG

SeTai ---SLLARQQ-----------TTPTTPLPL--------PNPNLRAYGPTARPRAPPP--PQMP----------PQRHHHSTLSNILTGTNHRANNMNGGG

DmTai ---KLLQNPPKIPKAPNPEQPLQVKTLPDI--------TSSTVSST------------------------LAAPGNLISAGSTGPKAAANR---------

TcTai ---SLLAKTP-----------NNQQPIPHI--------PVSLMSATPQDILPRISS--LPETV----KTSKPMPTRSTTSVTQQRTVVKNTLPDNMSRMG

LmTai ---SLLAKEP-----------SQPATIPPI--------PASVISATPQEILPRVSK---PQTPSW--PSGNAQPPSSTAAPSRPTQQVTDVSRQQVRPQS

AmTai ---SLLAKQP-----------PQPTTIPPI--------PASVISATPQDKLVRIGL-----------KSQQPQPQTQSQS--------------------

xLL

AiTai VNVS----GEGMGNMGASHLQQVLQSGGLAGGAR------------------------------------------------------------------

HaTai GVPA----EGGGAALGSSHLQMVLQSGALAAGARY------------------PA---PHAMQHY----------PQQAPGHVYNNQPQQ----------

SeTai GVSSVGTEGPAVSMGNSSHLQMVLQSGGLAGGSRY----------------PAPA---PHPLHYS-----APAPAPAPGPAPLYNNQP------------

DmTai -----------------NRKQQQQQQQQQQQQQQ-----QQVAGIPSQQQQQQPN--DVYLSQQQ---------------------------QQQLQ---

TcTai ARQP-----------TTNYLTTMLTHPN--------------------SMHHQRT---PDNRQMA-----QVDS--------------------------

LmTai NRQS-----------ATPFLNQMLTQQDHQQSNLLLQQSIQQQKVQQQQQQQQPS---PQQSQQQ-----QVQSSPQQAQHQVQQQQQQQQQQQQQQ---

AmTai -------------------QQQQQQQQQQQQQQQQQQQQQQQQQQQQQQQQQQPW---TGSSMQSVGGNNTITTTATSARTPLQTQSRQLPHRQTTNTYL

AiTai ---------------------------------SSN--RAQGG-GAGD------SEV--------------------PSDQV--LSDILDEVIENMPDA-

HaTai --------------------------------TTSNNVRPQGG-PAGD------SEV--------------------PSDLT--LSDILDEVIENMPDA-

SeTai ---------------------------------TSGNARPQGGSGSGD------SEV--------------------PSDQT--LSDILDEVIDNMPDA-

DmTai -SPQLAFQHQQL-----------------ATTATTSITTAASTSAAAAAAAAI--------LG--------------EGDSE--LSKLLDSVMEYYPDDT

TcTai -----------------------GSYTSPATSST---------DGNIDASMWVDNNN--------------------SNDPL--LSDILDQVMDIVPDE-

LmTai -QQQVQQQRAQL-------NQLDSSFNLPVSGTTTVDFRTAPGSGVSSPSTW--DNQ--------------------SSDPV--LSDLLDQVIDIVPDD-

AmTai THMLSQQQRPQM-------GQMDSEF------TSSGEYRQAS----TDLNTW--DNQ--------------------SSDPD--LSDILDQVIEFVPDE-

AiTai ------------------------DRQQPDVNVRQRQGKNEKNAMINAIRQ---SLMQYE-VGK-----SPGGSSPS-GGGAG-SAGGAG--AGPYS---

HaTai ------------------------DRPQPDVNVRQRQGMKEKNAMINAIRQ---SLMQYEAVGK-----SPGGSSPSAGAGPG-VAAGAAPRAAPAG---

SeTai ------------------------DRPPPDVSVRQRQGMKEKNAMINAIRQ---SLMQYEAVES-SP--RPGGSSPGGGSAAGPVSAGSA-----YS---

DmTai PIVTNAPSEASAINDIQKSLMLDVESAAFG-NDLNQQLMMTQQQ-QHQQQQQQQQLLALQLAQ------QQQQQRQQHLQQPP-AYPGML----NMQ-QQ

TcTai ------------VGR-----TFSAGEGQQP-NNFHPNELSEKMA-INIIQK---SLMQCESVVK-----SPSSPTITLPGTPP-AYTPAA-----MT---

LmTai ---------SSAIMN-----MLDAMESSPN-NSGFQQGLNEKMA-IHAIQK---SLMLCESAVK-----SPTSPTVSLPGTPP-TYAAST-----MS---

AmTai -----AITDSSAIAN-----LLDVTEAPQN------NAMNETMA-INAIQK---SLMLCETAV------NPTSSTITIPSTPP-AYSTAL-----GT---

AiTai -VQSPVSPGSP-------------------EPRERWRALQCG--S------------------------------------LYEPARAR-QHAEQQRARL

HaTai -YGAPVSPGSP-------------------EPRERWRALQCGLPG------------------------------------YADPARAR-ALVEQERARL

SeTai -VASPVSPGSP-------------------ELRERWRGGPAPGPA--------AGAP------------------------YSDTARAR-ALVEQQRARL

DmTai QHQQQQQQNQQHIMQRLEAMRNQGNQGFQRPPPMY---------------PARGRGPMNAV------ATPGGVVLPAQQQLRNIRQQQQ-LAAAQQKERL

TcTai -TQNSQMRPFQ-------------------PPPNYNQASFI----AKQRLAVRPGAPQYAV------ATGGL---TNSQQ-----LQIQ-RRQQEEKRRL

LmTai -NQTPQQGNFP-------------------PPPVYQRPTRFGLPAGTTPVPGRPGAPQYGIGVLNQQALGSG---VLIQQ--ALIQQRKILQQQQQKQRL

AmTai -TPVTTSHSYQ-------------------PPPMYQQQPRM-------RFNTQPGIRQTTAQFTQQQQL------QLQQQRTKLIQQQQ-QQQQQLKQRL

AiTai LQMQRSQQMLVSPEAAE--------------------QPQPDLGSTINALVSATPPNVALTRTDYHHPIYHQNNQMGPNYGTNKITTSQ-----------

HaTai LQLQRTQQMLVSPEAAE--------------------QPQADLGSTINALVSATPPNVALTRTDYHHQMYHQNNQMGPNYGTNKITTSQ-----------

SeTai LQLQRSQQMLVSPEAAE--------------------QPQADLGSTINALVSATPPNVALTRTDYHHQIYHQNNQMGPNYGTNKITTSQ-----------

DmTai LQQQQKQQLLVPENASEYWGNSNLQLIIKLIVSYILAGMNAGLNNIGSLLNTTGAPNVSLSRTNL-----PSDAQLSPNFAQT-LMQQQ--LSPG--RSA

TcTai LQQQQQQELLIPSNATA-------------------AEINSGLQNIDSLLNNTVAPNVSLQRSASL----P-ESQLSPNYGGQ-LNQPNQRIN----SQQ

LmTai LQQQQQQQLLIPSNATA-------------------DQLPTGLQNIDSLLNNTVAPNVSLQRSASV----PADPQLSPNYGGQILNSAASQISPGRLNQQ

AmTai LQQQQQQQLLIPSNATA-------------------PEQITTIHNIDNLLNNTVAPNVSLQRSSV-----P-DSQVSPGYGGS-VQMPS-----GHRLAH

AiTai ----------------------------------------------------------------------------------------------------

HaTai ----------------------------------------------------------------------------------------------------

SeTai ----------------------------------------------------------------------------------------------------

DmTai PYSPQPN---------------------------------------------------------QGYA-----------------PQFPQPGQRLSPQQQ

TcTai PYSPHSQL-VSPIGQQA------------------------------------------------GFP-------------QTSAANYQQAGARLS----

LmTai PYSPHSQL-ASPLGQQQVFSTVSTGINSFNSGQQAAQSRLSPHPPTGLPSFQQSQLSPRISQGQPGYPVLTQGTLAQSPQGQQTVSSWSQAASRLSLQQQ

AmTai SYSHPSTLPQHPIVNSN-----------FNSGQQ------------------------------------------------------VSAAARLS----

AiTai ----------------------------------------------------------------------------QNPMLSRQLSVSING---------

HaTai ----------------------------------------------------------------------------QNPMLSRQLSVAGSGAYSHSG---

SeTai ----------------------------------------------------------------------------QNPMLSRQLSVSGAG-YSH-----

DmTai QQLSQQQQNNVQQQQLAYQQQQVGDGGRSNTPFGSNSGMQSPGMQNSPQQWGSGGGGGGGPGGPLPSGNAGRTLQQHNPMLIAQLQGV--SPY-------

TcTai PQFTQQMALRQAYPQGSAQGQN-------------------------WQQ------------------NQAR-LSLQNPMLNAQLTG----NY-VSGRNF

LmTai QQQQQQQQQQQQQQQQQQQQQQ-------------------------QQQ------------------QQQQ-QQQQNPVLNAQLSG----NYGTAGRPF

AmTai PHSSAGI-LSFSHPQ---------------------------------------------------------------PLSPRVTQG----NYGNTPRLF

AiTai --GVS-----------------------PALHAPMSPAPQP---------------------------------YHRAPRPHLVGGYYEDAG--------

HaTai --GVSTHT-------------------AAALHTPMTPA-QP---------------------------------YHRPPRPHLVGGYYEEGG--------

SeTai ---------------------------GASLHTPG--A-QP---------------------------------YHRAPRPHLVGGYYEEGG--------

DmTai --------------NARQYQQNQR----RGLNSPG--AVGPGGNPAAQAALQRQNSFQGQGGGGATTPDGSGVGFGGPQSPYGTNV--------NVFQQQ

TcTai QRAPNQPSAAQQ----QQQQQQQQ----RSLNSPG--TVTS-----------RHSPYQQDS-------------FPPPSSPNSTAF-----------NQT

LmTai --APQRPQQQQPS---QQQQQPQQLPPVRSLASPGGAVVGP-----------RQSPYQAEQ-------------FPPPSSPGTTTGFQAQG------QFP

AmTai --TVNQVRTQQQS-TAQQQLQQQQ----RSMPSPG-TPASA-----------RQSPFPAET-------------FPPPTSPTATQF--PPGPNPGAPNPS

AiTai ------------------AGAAGYCGEYA-----------------------------RCGPLGPHA-----------PHA-------------------

HaTai ------------------AGAAGYCGDYA-----------------------------RCGPPHPHP--------HPHPHA-------------------

SeTai ------------------AGAAGYCAEYA-----------------------------RCAALGAHA------PLAPLHHA-------------------

DmTai QLQRLQRQGSVPQATQHLPGSPRFGSSPGSSNNTDSSAAAGNQQQQQQQQQQQ----QQQHQQQQHQQLMNGLSMSPHPH--------------------

TcTai QYLRLQRANSVPTATTQLPGGLGSPRPYG-----------------------------REHHPHPYP------PIPPNPH--------------------

LmTai QQLRLQRTISAPSATTQLPGGVNHSRHYGTKQELPHPALSPSEPVSPHPFHPHGPPLPDPHHHHPHP------LSHPHPHHHHHHIPHHQQQPQPQHHNP

AmTai AQYRLQRTTSTPSATTQLPGGVGSPRHYGGVSK-EQPLLSPS---------------------HPHSGCN---PATPTHN--QHNVTNTQQHFSNQQHS-

AiTai -----PHA---ALD-----HQHACAGNGGIVSGAG---GPG--GPVGP----------GGPP-------------GGAG-PAGG--TSEYVRNEL-RAVV

HaTai -----PHP---TLD-----H-HACAGNGG----------------------------------------------GGAG--AGG--TSEYVRNEL-RAVV

SeTai -----PHPHASPLD-----HQHACAGNGGGVSGSGGVAGVG--GVAGV----------GGVA-------------GVAGGGSGG--TSEYVRNEL-RNMV

DmTai -----PHPAMMGVG-----GMGSGGGNGIIGLGGG---GGNYGATLGGYGQSQQANDFYGRA-QTA---------GGGGSVAGGNANSEFVKQEL-RAVV

TcTai -----QHPMMYQQD-----SSQYCYDQTGLQLAYN---GAD-----------------RGRA-PPHL---------QAGVSASG-PTSEFVRQEL-RAVV

LmTai HQPSQHHPVMYQAQPRPPPDSQFCYDQPNFQL-YG---ASE-----------------RGRP-QAHTPTNHQPGGGAGGGGNGG-MTSEYVRQEL-RAIV

AmTai ---SMIYHTANTINTADMQNNQFCYDRTTVPLYSS---GPG---------DTQ-----DARPLPPGNPVNH-----QLGGNASS--TSEFVRQELRRAIV

AiTai GARS--------------------ARPELHPLQPP---DMDSL-MS--FDMT-----PPGSYK-------------------------------------

HaTai GAR---------------------ARPELHALQPP---DMDAL-IA--YDIT-----PPEYYGGGLGGGGAGGGR-------------------------

SeTai -SR---------------------ARPDLHPLQAP---DMDPL-MS--FDMT-----PPG-------GGSVVGGRATSASTNSWESQQSTPSTTEAGSAE

DmTai SVRAQQAAAAATGGAGVGGGVAQRGQTPQSPLQQGS-VIGGGGGIVGGFNST--N--TMG-----------------NVTPTGSGNVSNSMLNTPPDPTL

TcTai GARTGQAQTAP-----------NRTQSQLLNQQQV---DLEALGIT--FEMP-----TSG----------------ASDSPKLWGAMGSDMGSMSPQP--

LmTai GARQSNGGSSGSVGMAVSG---QQRQLQLGPGQSVSAADLEALGFV--CEPP-----STG----------------TSDSPKLWGGIGSDLSSGSPQAGF

AmTai GARTQQQQQQR---------IPNNIQNNLSG--QVSQDDLEALGLT--FEMS-----SAGE-------------AVVSDGPAKSWAIGSTGSAP------

AiTai ------------------------------LIR

HaTai --------------------------------- ID:81.7%

SeTai AGSAADGDEARAGPAGGAAAAAKASLLQKLLSQ ID:75.4%

DmTai S-----------------------------FSF ID:17.4%

TcTai ATSRSSMEEARA----GDHGNKSSSLLQKLLSE ID:24.0%

LmTai SSSRNPTEESPR------PGDHKSSLLQKLLSE ID:20.0%

AmTai SSSRTSMEEVAR----GDPKVNQSSLLQKLLSE ID:20.6%
